# Transcriptome-Based Screening of Candidate Low-Temperature-Associated Genes and Analysis of the *BocARR-B* Transcription Factor Gene Family in Kohlrabi (*Brassica oleracea* L. var. *caulorapa* L.)

**DOI:** 10.3390/ijms25179261

**Published:** 2024-08-27

**Authors:** Shuanling Bian, Dengkui Shao, Qingsheng Zhao, Quanhui Li, Yanjing Ren

**Affiliations:** 1 Academy of Agriculture and Forestry Sciences, Qinghai University, Xining 810016, China; bianshuanling2024@163.com (S.B.); 2006990015@qhu.edu.cn (D.S.); liquanhui_2008@163.com (Q.L.); 2 Laboratory of Research and Utilization of Germplasm Resources in Qinghai-Tibet Plateau, Qinghai University, Xining 810016, China; 3 Key Laboratory of Germplasm Resources Protection and Genetic Improvement of the Qinghai-Tibet Plateau in Ministry of Agriculture and Rural, Xining 810016, China; 4 College of Agriculture and Animal Husbandry, Qinghai University, Xining 810016, China; zhaopunkwave@outlook.com

**Keywords:** kohlrabi (*Brassica oleracea* L. var. *caulorapa* L.), low-temperature stress, metabolites networks, differentially expressed genes, *BocARR-B* transcription factor, gene relative expression level analysis

## Abstract

Low temperature is a significant abiotic stress factor that not only impacts plant growth, development, yield, and quality but also constrains the geographical distribution of numerous wild plants. Kohlrabi (*Brassica oleracea* L. var. *caulorapa* L.) belongs to the Brassicaceae family and has a short growing period. In this study, a total of 196,642 unigenes were obtained from kohlrabi seedlings at low temperatures; of these, 52,836 unigenes were identified as differentially expressed genes. Transcription factor family members *ARR-B*, *C3H*, *B3-ARF*, etc. that had a high correlation with biochemical indicators related to low temperature were identified. A total of nineteen *BocARR-B* genes (named *BocARR-B1*–*BocARR-B19*) were obtained, and these genes were distributed unevenly across seven chromosomes. Nineteen *BocARR-B* genes searched four conserved motifs and were divided into three groups. The relative expression level analysis of 19 *BocARR-B* genes of kohlrabi showed obvious specificity in different tissues. This study lays a foundation and provides new insight to explain the low-temperature resistance mechanism and response pathways of kohlrabi. It also provides a theoretical basis for the functional analysis of 19 *BocARR-B* transcription factor gene family members.

## 1. Introduction

Kohlrabi (*Brassica oleracea* L. var. *caulorapa* L.) is a member of the Brassicaceae family, and it has a short growing period [1]. Kohlrabi is mainly cultivated in Europe, the United States, and Canada [2]. The typical biological feature of kohlrabi is its fleshy, swollen stem (bulb), which provides a high nutritional value for humans. Kohlrabi contains plenty of vitamin C, glucosinolates, and mineral nutrients such as potassium [3,4,5,6]. The swollen stem of kohlrabi is commonly green, greenish-white, or purple and is mainly consumed by humans as raw, cooked, or preserved [7,8].

Low temperature is one of the important abiotic stresses that not only affect plant growth, development, yield, and quality but also limit the geographical distribution of many wild plants [9,10]. There are two types of low-temperature stress, namely cold stress (0 °C~16 °C) and freezing stress (<0 °C). Generally, the adaptability of plants to low temperatures above the freezing point is called cold resistance, and the adaptability to low temperatures below the freezing point is called freezing resistance [11]. Due to its variation among species and different low-temperature severities, physiological and gene network responses to low temperatures are complex; thus, temperate plants may be more cold-tolerant than tropical plants [12]. Therefore, studying the response mechanism to cold stress and excavating the differentially expressed genes in different plants are very important. Using the transcriptome sequencing approach, screening differentially expressed genes (DEGs) in response to adversity stress is a technical means that has been reported several times [13,14]. Li et al. [15] reported that multiple transcription factors, including the *bHLH*, *MYB*, *HD-ZIP*, and *ERF* families, were holistically elevated in tall fescue under low-temperature stress. Dai et al. [16] reported that the cold tolerance of *A. thaliana* transgenic plants was enhanced due to the overexpression of *OsMYB4* and that *OsMYB3R-2* from Oryza sativa influenced the expression of multiple genes in the cold-response pathway. Yang et al. [17] found that 33 TFs from six families of *AP2/ERF*, *MYB*, *WRKY*, *GRAS*, *bHLH*, and *bZIP* were closely associated with the response to low-temperature stress in poplar (*Populus tomentosa*). However, the response mechanism of kohlrabi under low-temperature stress has not been reported.

The response of plants to abiotic stress can lead to changes in the variety and number of metabolites [18,19]. The metabolic network response mechanism of plants has been widely studied under low-temperature stress. Thomashow [20] found that the encoded proteins of *CBF* genes can activate the expression of *COR* genes, resulting in the accumulation of protective substances in Arabidopsis. Zhang et al. [21] showed that the sucrose content was increased by the overexpression of *SlHSP17.7* in the hexose-accumulating Moneymaker tomato. Kidokoro et al. [22] reported that the expression of *CBF1* and *CBF2* was regulated by *CAMTA3* and *CAMTA5* in response to rapidly decreasing temperatures. However, the molecular and metabolic network mechanisms of kohlrabi’s response to low-temperature stress have not been reported.

The transcription factor gene family of type-B authentic response regulators (*ARR-Bs*) is an important plant-specific transcription factor family involved in positive regulators of cytokinin signal transduction, and it plays important roles in abiotic stress resistance and plant development in, at least, the typical PF00072 and PF00249 domains [23,24,25]. Nguyen et al. [26] found that *ARR1*, *ARR10*, and *ARR12* play key roles in cold and drought signaling in Arabidopsis. Jeon et al. [27] reported that *type-B ARRs* can regulate *type-A ARRs* that improve cold tolerance in Arabidopsis. Kang et al. [28] showed that cold stress resistance can be improved by the overexpression of *ARR22*. All in all, *type-B ARRs* play a direct or indirect key role in positively responding to environmental stress. The *ARR-B* family has been researched in a variety of plants, mainly soybean [25], peach [29], tomato [30], and tea plants [31]. The identification and analysis of the *ARR-B* transcription factor family in kohlrabi could provide a theoretical basis for distinguishing the function of the *ARR-B* gene.

For this study, we first analyzed the DEGs in kohlrabi under low-temperature stress. Second, the differentially expressed transcription factors related to low temperature were screened. Meanwhile, we analyzed the *BocARR-B* transcription factor gene family. This study laid a foundation and provided new insight into the low-temperature resistance mechanism of kohlrabi. It also provided a theoretical basis for the functional analysis of family members of the *BocARR-B* transcription factor gene.

## 2. Results

### 2.1. Biochemical Indicators Changes in Kohlrabi under Low-Temperature Stress

**Figure 1:**
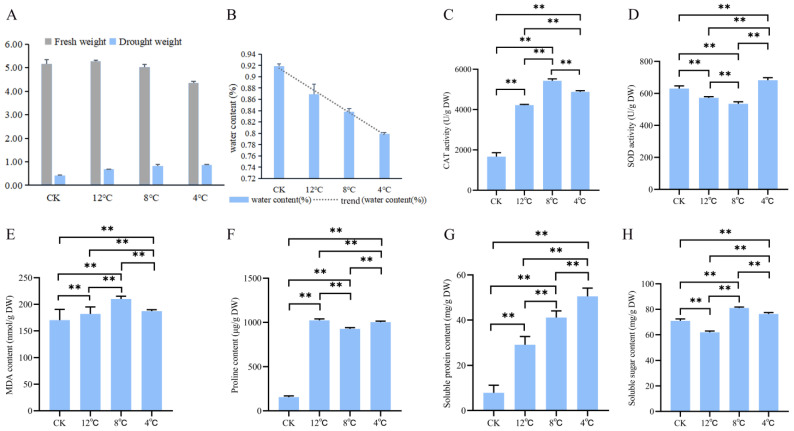
Biochemical indicators of kohlrabi seedlings at low temperature (CK) and under low-temperature stress at 12 °C, 8 °C, and 4 °C. (**A**) Fresh and dry weight. (**B**) Water content. (**C**) Catalase (CAT) activity. (**D**) Superoxide dismutase (SOD) activity. (**E**) Malondialdehyde (MDA) content. (**F**) Proline (Pro) content. (**G**) Soluble protein content. (**H**) Soluble sugar content. ** Represents a significant difference when the *p*-value is 0.01.

We initially conducted measurements of the changes in both the fresh and dry weights of kohlrabi seedlings under low-temperature stress using fresh kohlrabi seedlings (fresh weight (FW)) that were first weighed. Subsequently, fresh kohlrabi seedlings were dried at 90 °C for 10 h (dry weight (DW)) (Figure 1A). Then, the water content was calculated using the following formula: water content = (FW − DW)/FW*100%. The results revealed a decreasing trend in the water content of kohlrabi seedlings, with values of 91.88%, 86.93%, 83.82%, and 79.86% recorded at temperatures of CK (22 °C), 12 °C, 8 °C, and 4 °C respectively (Figure 1B). Then, six biochemical indicators of change were determined, including catalase (CAT) activity, superoxide dismutase (SOD) activity, malondialdehyde (MDA) content, proline (Pro) content, soluble protein content, and soluble sugar content. Among them, all biochemical indicators (fresh weight) were significantly higher in kohlrabi seedlings under low-temperature stress than those in untreated kohlrabi seedlings (CK) (Table S1). To determine the severity of the stress treatment, all biochemical indicators were normalized by dry weight. CAT activity was significantly stronger after treatment than that in the control and significantly weaker at 4 °C (4881.14 ± 58.09 U/g DW) than at 8 °C (5427.04 ± 94.38 U/g DW) (Figure 1C). SOD activity was significantly weaker at 12 °C (572.01 ± 7.60 U/g DW) and 8 °C (534.81 ± 12.67 U/g DW) after treatment than that in the control and significantly stronger at 4 °C (682.06 ± 16.50 U/g DW) than that in the control (Figure 1D). MDA content was significantly higher after treatment than that in the control and significantly lower at 4 °C (187.23 ± 2.66 nmol/g DW) than those at 8 °C (210.19 ± 5.09 nmol/g DW) (Figure 1E). The proline content was significantly higher after treatment than that in the control and significantly lower at 8 °C (927.00 ± 13.53 μg/g DW) than those at 12 °C (1023.18 ± 16.73 μg/g DW) and 4 °C (1003.75 ± 10.90 μg/g DW) (Figure 1F). The soluble protein content was significantly increased after treatment; the content was 7.86 ± 3.35 mg/g DW at CK and 50.53 ± 3.62 mg/g DW at 4 °C (Figure 1G). The soluble sugar content was significantly lower at 12 °C (61.93 ± 1.00 mg/g DW) after treatment than that in the control and significantly higher at 8 °C (81.05 ± 0.77 mg/g DW) and 4 °C (76.38 ± 1.10 mg/g DW) than in the control (Figure 1H).

### 2.2. Transcriptome Data Summary and KEGG Enrichment Analysis

To understand the molecular basis of kohlrabi seedlings under low-temperature stress, twelve samples (CK-1, CK-2, CK-3, 12 °C-1, 12 °C-2, 12 °C-3, 8 °C-1, 8 °C-2, 8 °C-3, 4 °C-1, 4 °C-2, and 4 °C-3) were subjected to total RNA extraction and transcriptome sequencing. A total of 51,903,722~43,469,990 raw reads were obtained, with an average of 48,007,155 raw reads. After filtering the adaptor and low-quality sequence, a total of 48,868,442~42,735,880 clean reads were obtained, with an average number of clean reads per sample of 45,754,446. The guanine and cytosine bases’ content ranged from 43.97 to 47.42%, the Q30 value for each library was >93.33%, and the Q20 value for each library was >97.67%.

**Figure 2:**
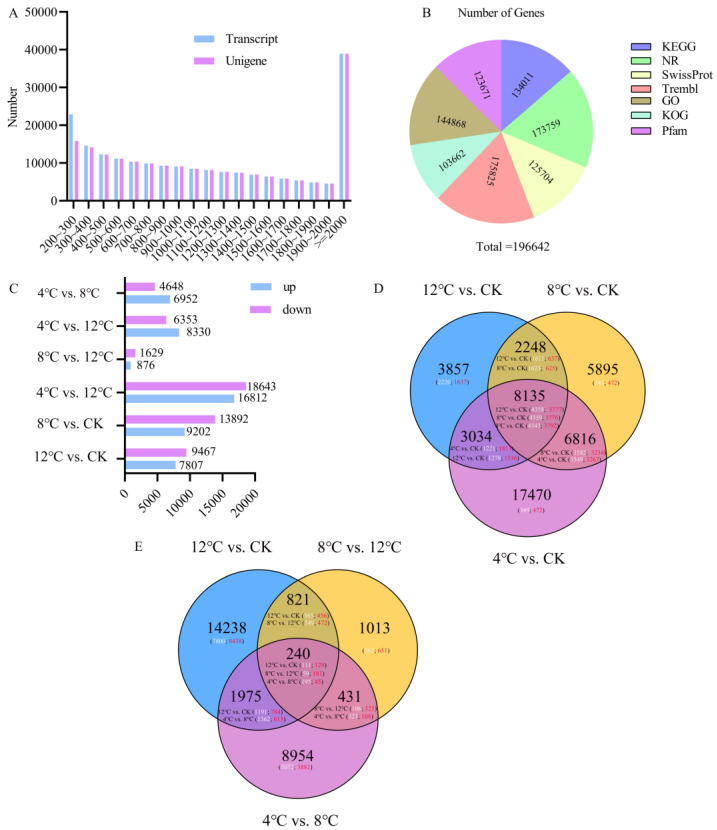
Transcriptome data summary and analysis of differentially expressed unigenes in kohlrabi seedlings under low-temperature stress. (**A**) The transcript and unigene sequences’ length distribution. (**B**) Functional annotation of assembled unigenes. (**C**) Differentially expressed unigenes in different pairwise comparisons. (**D**) Venn diagram of differentially expressed unigene numbers in pairwise comparisons between control and treatment of 12 °C vs. CK, 8 °C vs. CK, and 4 °C vs. CK. White and red digits indicate the number of downregulated and upregulated genes, respectively. (**E**) Venn diagram of differentially expressed unigene numbers in pairwise comparisons of treatment of 12 °C vs. CK, 8 °C vs. 12 °C, and 4 °C vs. 8 °C. White and red digits indicate the number of downregulated and upregulated genes, respectively.

The size and distribution of the transcripts’ and unigenes’ length showed that 49,843 (24.39%) transcripts were less than 500 bp in length, and 38,951 (19.06%) transcripts were greater than 2000 bp. Similarly, a total of 42,181 (21.45%) unigenes were less than 500 bp in length (Figure 2A). A total of 196,642 (100.00%) unigenes were annotated in the Kyoto Encyclopedia of Genes and Genomes (KEGG), NCBI non-redundant (NR), Swiss-Prot, Gene Ontology (GO), euKaryotic Ortholog Groups (KOG), Trembl, and Pfam databases. The results showed that 134,011 (68.15%), 173,759 (88.36%), 125,704 (63.93%), 175,825 (89.41%), 103,662 (52.72%), 144,868 (73.67%), and 123,671 (62.89%) unigenes were annotated in these seven databases, respectively (Table S2). Furthermore, 177,301 (90.16%) unigenes were assigned to be annotated in at least one database (Figure 2B). RNA-Seq results showed that a total of 52,836 unigenes were identified as differentially expressed genes. Then, we performed pairwise comparisons of all DEGs. In the comparison of 12 °C vs. CK, 17,274 DEGs (7807 upregulated and 9467 downregulated at 12 °C) were identified; in the comparison of 8 °C vs. CK, 23,094 DEGs (9202 upregulated and 13,892 downregulated at 8 °C) were identified; in the comparison of 4 °C vs. CK, 35,455 DEGs (16,812 upregulated and 18,643 downregulated at 4 °C) were identified; in the comparison of 4 °C vs. 12 °C, 14,683 DEGs (8330 upregulated and 6353 downregulated at 4 °C) were identified; in the comparison of 4 °C vs. 8 °C, 11,600 DEGs (6952 upregulated and 4648 downregulated at 4 °C) were identified; and in the comparison of 8 °C vs. 12 °C, 2505 DEGs (876 upregulated and 1629 downregulated at 8 °C) were identified (Figure 2C).

At the same time, the 8135 common DEGs were analyzed in the comparison of 12 °C vs. CK, 8 °C vs. CK, and 4 °C vs. CK (Figure 2D, Table S3). Similarly, 240 common DEGs were searched for in the comparison of 12 °C vs. CK, 8 °C vs. 12 °C, and 4 °C vs. 8 °C (Figure 2E, Table S4). The results of the KEGG enrichment analysis are shown in Figure S1, and the top three pathways in the KEGG enrichment analyses are demonstrated for 4 °C vs. CK (Figure S1A,B). The top three pathways are the metabolic pathway (ko01100), plant hormone signal transduction pathway (ko04075), and the plant–pathogen interaction (ko04626) pathway. The top three pathways in the KEGG enrichment analysis were demonstrated at 8 °C vs. CK (Figure S1C,D), with the top three pathways being the metabolic pathway (ko01100), biosynthesis of secondary metabolites pathway (ko01110), and the plant hormone signal transduction pathway (ko04075). Similarly, the top three pathways in KEGG enrichment analysis were demonstrated at 12 °C vs. CK (Figure S1E,F), with the top three pathways being the metabolic pathway (ko01100), biosynthesis of secondary metabolites pathway (ko01110), and the plant hormone signal transduction pathway (ko04075). K-means cluster analysis showed that ten subclasses were present. Subclass 5 included 4744 unigenes with a continuous decrease in expression profile from CK to 4 °C. Subclass 9 included genes with a continuous increase in expression from CK to 4 °C (Figure S2, Table S5). According to sequence alignment with the NR database, we found that Brassica napus had the most unigene hits (72,081, 41.48%), followed by *Brassica oleracra* var. *oleracra* (66,448, 38.24%) (Figure S3).

### 2.3. Availability Analysis of Transcriptome Data

**Figure 3:**
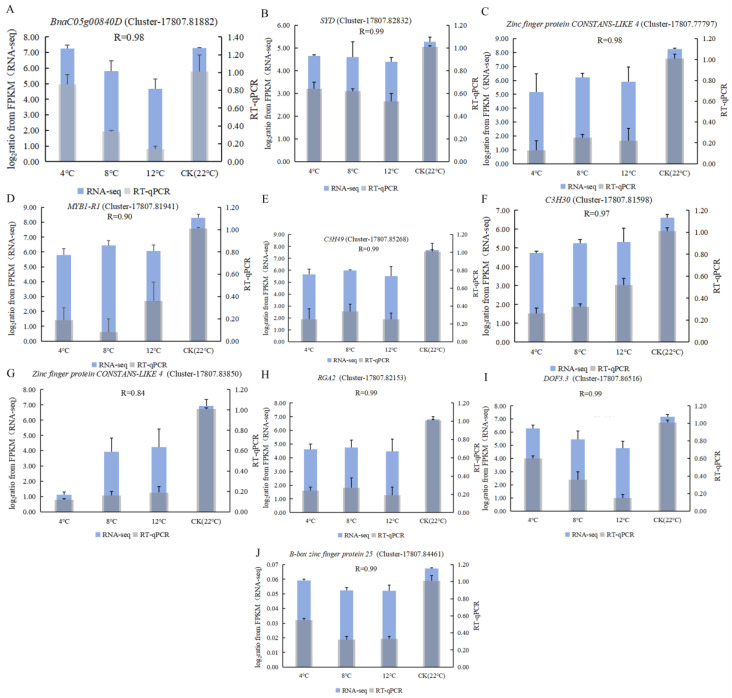
Availability analysis of transcriptome data using RNA-Seq and RT-qPCR was used to construct the expression-level bar graphs at 4 °C, 8 °C, 12 °C, and CK (22 °C). (**A**) *BnaC05g00840D* (Cluster-17807.81882); (**B**) *SYD* (Cluster-17807.82832); (**C**) *Zinc finger protein CONSTANS-LIKE 4* (Cluster-17807.77797); (**D**) *MYB1-R1* (Cluster-17807.81941); (**E**) *C3H49* (Cluster-17807.85268); (**F**) *C3H30* (Cluster-17807.81598); (**G**) *Zinc finger protein CONSTANS-LIKE 4* (Cluster-17807.83850); (**H**) *RGA2* (Cluster-17807.82153); (**I**) *DOF3.3* (Cluster-17807.86516); (**J**) *B-box zinc finger protein 25* (Cluster-17807.84461).

The accuracy of RNA-Seq data was confirmed via RT-qPCR. Ten randomly selected DEGs were tested in kohlrabi with three replications. The 2^−ΔΔCT^ of RT-qPCR and log_2_(FPKM) of RNA-Seq were analyzed at 4 °C, 8 °C, 12 °C, and CK (Figure 3). The results showed that the relative expression levels of DEGs (*BnaC05g00840D* (Cluster-17807.81882), *SYD* (Cluster-17807.82832), *Zinc finger protein CONSTANS-LIKE 4* (Cluster-17807.77797), *C3H49* (Cluster-17807.85268), *C3H30* (Cluster-17807.81598), *RGA2* (Cluster-17807.82153), *DOF3.3* (Cluster-17807.86516), and *B-box zinc finger protein 25* (Cluster-17807.84461)) were highly consistent with RNA-Seq data (R > 0.95). In addition, the relative expression levels of DEGs (*MYB1-R1* (Cluster-17807.81941)) were consistent with RNA-Seq data (R ≥ 0.90). The results of the analysis indicated that RT-qPCR and transcriptome data had a significant positive correlation, which showed that transcriptome data were available.

### 2.4. Basic Genetic Analysis of Metabolite Networks under Low-Temperature Stress

**Figure 4:**
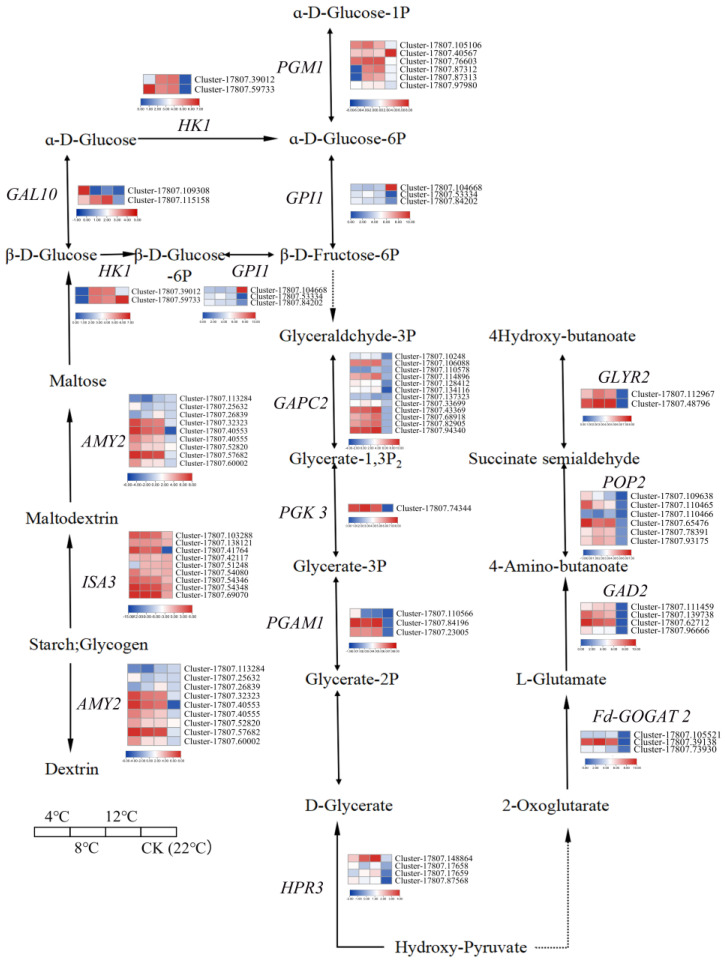
A putative interplay of kohlrabi seedlings under low-temperature stress. The DEG changes were represented by the log_2_FPKM. Blue and red, respectively, represent a decrease and an increase in the expression level of genes.

According to the literature and the web-based KEGG database, the metabolic pathways were proposed and constructed to achieve an in-depth understanding of the regulatory pathways that respond to low-temperature stress. The important known pathways include the Glycolysis/Gluconeogenesis (ko00010), starch and sucrose metabolism (ko00500), glyoxylate and dicarboxylate metabolism (ko00630), and the butanoate metabolism pathway (ko00650) (Figure 4). Sixty-six DEGs related to low temperature were identified in kohlrabi seedlings. In the Glycolysis/Gluconeogenesis pathway, four low-temperature-related DEGs (*PGM1*, *GPI1*, *GAPC2*, and *PGAM1*) were notably downregulated. In the starch and sucrose metabolism pathway, nine low-temperature-related DEGs (six *AMY2* and three *ISA3*) were notably upregulated or downregulated. In the glyoxylate and dicarboxylate metabolism pathway, no low-temperature-related DEGs were found to be notably upregulated or downregulated. In the butanoate metabolism pathway, five low-temperature-related DEGs (three *GAD2* and two *POP2*) were notably downregulated.

### 2.5. Analysis of Differentially Expressed Transcription Factor and Weighted Gene Co-Expression Network Analysis between Biochemical Indicators Related to Low Temperature and Differentially Expressed Transcription Factors

**Figure 5:**
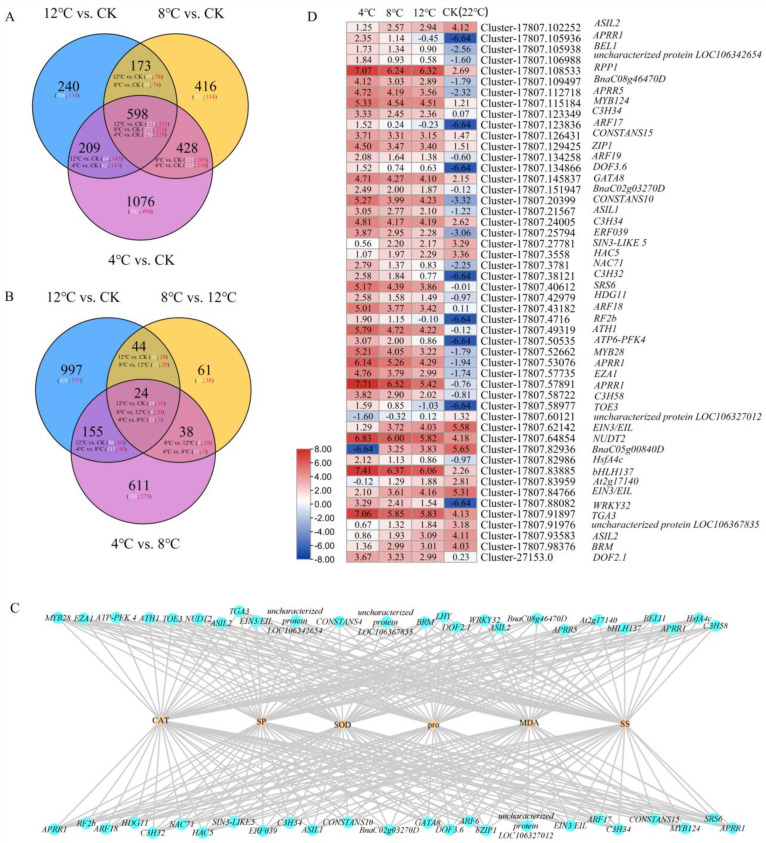
Analysis of differentially expressed transcription factors and identification of low-temperature-related differentially expressed transcription factors. (**A**) Venn diagram of differentially expressed transcription factors in the comparison of 12 °C vs. CK, 8 °C vs. CK, and 4 °C vs. CK. White and red digits indicate the number of downregulated and upregulated genes, respectively. (**B**) Venn diagram of the differentially expressed transcription factor in the comparison of 12 vs. CK, 8 °C vs. 12 °C, and 4 °C vs. 8 °C. White and red digits indicate the number of downregulated and upregulated genes, respectively. (**C**) Co-expression network map between differentially expressed transcription factors and CAT activity, SOD activity, MDA content, Pro content, and soluble sugar content. Yellow squares indicate biochemical indicators related to the low temperature. Blue circles indicate differentially expressed transcription factors. (**D**) Expression profile of differentially expressed transcription factors’ related low-temperature stress in kohlrabi using RNA sequencing. The changes in the differentially expressed transcription factors are represented by the log_2_FPKM.

To identify the differentially expressed transcription factors in kohlrabi under low-temperature stress, we performed pairwise comparisons of all differentially expressed transcription factors, with the results showing that 598 common differentially expressed transcription factors were searched for in 12 °C vs. CK, 8 °C vs. CK, and 4 °C vs. CK (Figure 5A, Table S6). Similarly, a total of 24 common differentially expressed transcription factors was searched for in 12 °C vs. CK, 8 °C vs. 12 °C, and 4 °C vs. 8 °C (Figure 5B, Table S7). Biochemical indicators (CAT activity, SOD activity, MDA content, pro content, soluble protein content, and soluble sugar content) and differentially expressed transcription factors were analyzed using weighted gene correlations. Fifty differentially expressed transcription factors showed a high correlation with CAT activity, SOD activity, MDA content, pro content, soluble protein content, and soluble sugar content, including *WRKY32*, *bHLH137*, *APRR1*, *MYB28* and so forth (Figure 5C). In order to further apprehend the trend in expressions of these differentially expressed transcription factors, a heatmap of the expression of 50 of these factors was constructed using their Log_2_FPKM values (Figure 5D). The results showed that the expression level of eleven differentially expressed transcription factors (*ASL2*, *SINS-LIKE5*, *HAC5*, *uncharacterized protein LOC106327012*, *two EIN3/EILs*, *BnaC05g00840D*, *At2g17140*, *TGA3*, *uncharacterized protein LOC106367835*, *ASIL2*, *BRM*) have a decreasing trend from CK to 12 °C, 8 °C, and 4 °C. Then, 50 transcription factors show an increased trend from CK to 12 °C, 8 °C, and 4 °C. Then, 50 transcription factors were classified via the transcription factor annotation file, and the results showed that the family of 50 transcription factors contains four *Pseudo ARR-B* transcription factor family member genes, four *C3H* transcription factor family member genes, three *AP2/ERF* transcription factor family member genes, three *bZIP* transcription factor family member genes, and others.

### 2.6. Joint Analysis of Low-Temperature-Related Differentially Expressed Transcription Factors and DEGs

**Figure 6:**
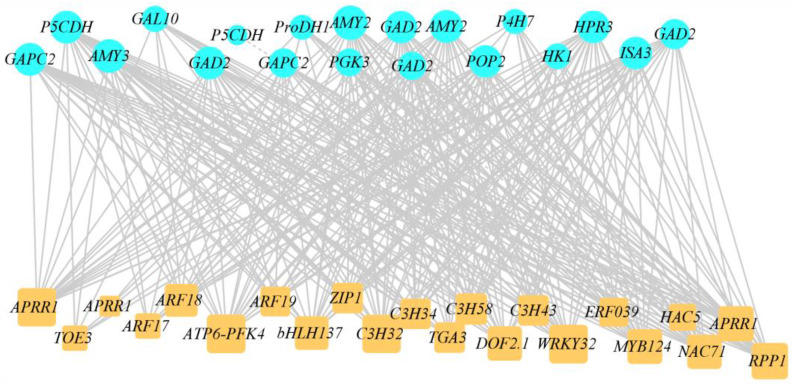
Co-expression network map between differentially expressed transcription factors related to the low temperature and DEGs related to the metabolites pathway. Blue circles indicate DEGs related to the metabolites pathway; yellow squares indicate differentially expressed transcription factors related to the low temperature.

To further accurately identify low-temperature-related DEGs and differentially expressed transcription factors, the interaction regulatory network between DEGs and differentially expressed transcription factors was analyzed using weighted gene correlations (Figure 6, Table S8). The results showed that nineteen DEGs showed a high correlation with low temperature, including *GAD2*, *AMY2*, *POP2*, *GAL10*, and others. Similarly, twenty-two differentially expressed transcription factors were obtained, including *APRR1*, *WRKY32*, *TOE3*, *bHLH137*, *ARF17*, *MYB124*, and others. We speculated that these twenty-two differentially expressed transcription factors may improve the low-temperature resistance of plants and thus promote the polymorphic aggregation of sugar and proline in the pathway. We hypothesized that these twenty-two differentially expressed transcription factors may regulate specific genes, thereby enhancing the synthetic accumulation of sugar and proline in the pathway and potentially improving low-temperature resistance. The twenty-two differentially expressed transcription factors contain four *ARR-B* transcription factor family member genes, four *C3H* transcription factor family member genes, two *B3-ARF* transcription factor family member genes, and others.

### 2.7. BocARR-B Transcription Factor Gene Family Member Identification and Chromosomal Localization

In consideration of the high correlation of many *ARR-B* transcription factor family member genes with low-temperature stress, we identified and analyzed the *BocARR-B* transcription factor family in kohlrabi based on transcriptomes. A total of 19 putative *ARR-B* family candidate genes were obtained by confirming the conserved domain (PF00072 and PF00249) of ARR-Bs. Because some genes have similar sequences, we further performed multiple sequence alignments using the DNAMAN software. The result showed that these genes are unique. To distinguish between *ARR-B* transcription factor family member genes, in this study, nineteen *BocARR-Bs* were named according to their order on the chromosomes. The length of BocARR-B proteins ranged from 376 to 680 amino acids (aa), and the molecular weight (MW) ranged from 41,499 Da to 74,038 Da. The theoretical isoelectric point (pI) ranged from 5.08 to 8.87. The aliphatic index of BocARR-B proteins ranged from 69.4 to 89.15. The length of the CDS sequences and other details of the 19 *BocARR-B* genes are listed in Table 1.

**Figure 7:**
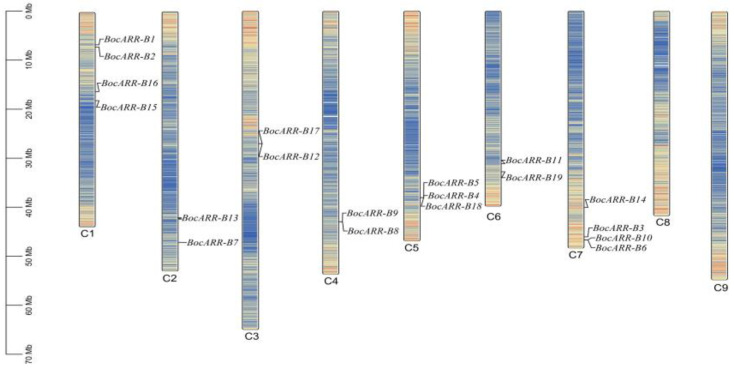
Chromosomal locations of *BocARR-B* genes in kohlrabi. Note: C1–C9 indicates nine chromosomes. The scale bar on the left shows the chromosome lengths (Mb). The blue and yellow regions of the chromosome represent a low and high gene density, respectively.

Nineteen *BocARR-B* genes were found to the chromosome (C) of kohlrabi. The results showed that nineteen *BocARR-B* genes were randomly distributed on the different chromosomes of kohlrabi, and no genes were distributed on the C8 and C9 (Figure 7). Chromosome 1 contained four genes (*BocARR-B1*, *BocARR-B2*, *BocARR-B3*, and *BocARR-B4*). Equally, chromosome 7 harbored four genes (*BocARR-B16*, *BocARR-B17*, *BocARR-B18*, and *BocARR-B19*). Chromosomes 2, 3, 4, and 6 harbored only two genes. Chromosome 5 contained three genes. This result highlights the irregular distribution of *BocARR-B* genes on the chromosomes.

### 2.8. Gene Structure and Conserved Motif Analysis of BocARR-B Transcription Factor Genes Family

**Figure 8:**
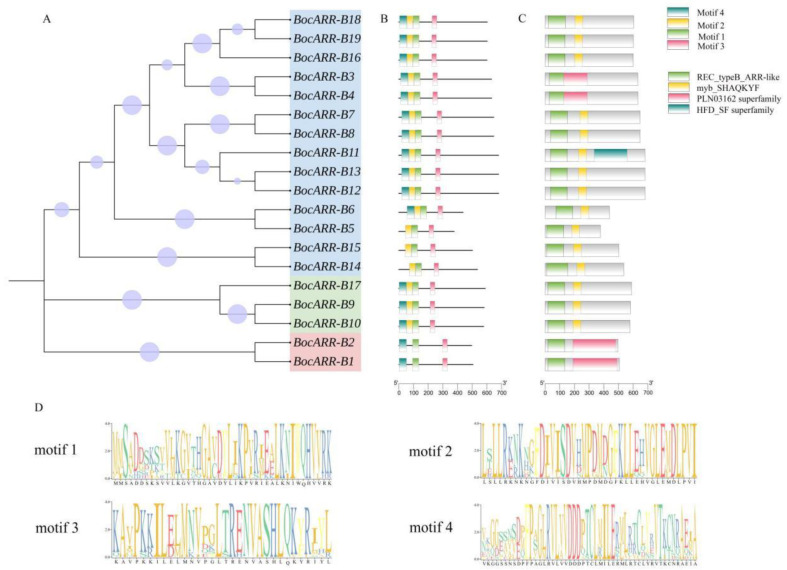
Evolutionary relationships, conserved protein motifs, and domains of the 19 *BocARR-Bs*. (**A**) Phylogenetic relationship analysis using amino acid sequences of *BocARR-Bs* by MEGA11. The size of the purple circle represents the bootstrap value. The display range indicates the branch metadata bootstrap. (**B**) Distribution of 4 motifs. (**C**) Distribution of REC_type_ARR-like domain, myb_SHAQKYF domain, PLN03162 superfamily domain, and HFD_SF superfamily domain. (**D**) Four different conserved motifs of *BocARR-Bs*.

To comprehensively comprehend the conserved traits of the BocARR-B protein and analyze its evolutionary variances, motif analysis was performed utilizing MEME, resulting in the identification of four types of conserved motifs (Figure 8D). Nineteen *BocARR-B* transcription factor gene family members were divided into three groups that contain group Ⅰ (*BocARR-B18*, *BocARR-B19*, *BocARR-B16*, *BocARR-B3*, *BocARR-B4*, *BocARR-B7*, *BocARR-B8*, *BocARR-B11*, *BocARR-B13*, *BocARR-B12*, *BocARR-B6*, *BocARR-B5*, *BocARR-B15*, and *BocARR-B14*), group Ⅱ (*BocARR-B17*, *BocARR-B9*, and *BocARR-B10*,) and group Ⅲ (*BocARR-B2*, and *BocARR-B1*) (Figure 8A). The results of motif analysis showed that motif 1 and motif 3 were present in all *BocARR-B* members, while motif 2 was absent in group Ⅲ, and motif 4 was absent in group Ⅰ in certain *BocARR-B* members (*BocARR-B5*, *BocARR-B15*, and *BocARR-B14*) (Figure 8B). An analysis of the conserved structural domains of the 19 BocARR-B proteins indicated that all BocARR-B proteins exhibited highly conserved structural domains. The distribution of the REC_type_ARR-like domain, myb_SHAQKYF domain, PLN03162 superfamily domain, and HFD_SF superfamily domain are shown in Figure 8C.

### 2.9. Evolutionary Analysis of BocARR-B Transcription Factor Gene Family

**Figure 9:**
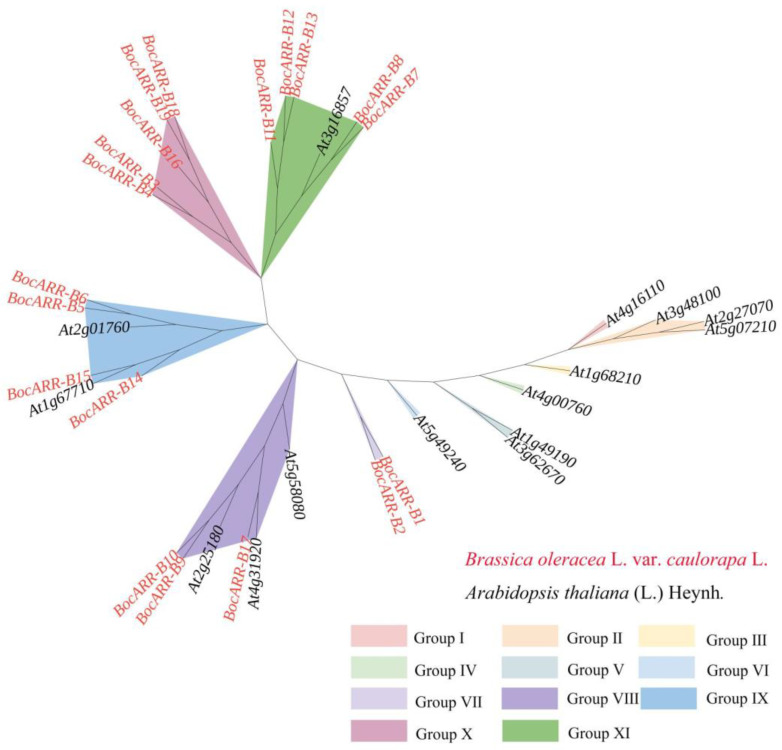
Evolutionary tree of *BocARR-B* family genes in kohlrabi and Arabidopsis.

To investigate the phylogenetic relationship of ARR-B proteins in kohlrabi, we generated a phylogenetic tree using the maximum likelihood (ML) method based on the multiple sequence alignment of 15 ARR-B proteins from Arabidopsis and 19 ARR-B proteins from kohlrabi (Figure 9). All the *ARR-B* genes were clustered into 11 groups named I–XI. Group I, group II, group III, group IV, group V, and group VI contain only *ARR-B* genes from Arabidopsis. All the *ARR-B* genes were clustered into 11 groups named I–XI. Group VII and group X contain only *ARR-B* genes from kohlrabi. The *ARR-B* genes from kohlrabi were clustered into group IX, group X, and group XI.

### 2.10. Expression Analysis of BocARR-Bs via RT-qPCR and RNA Sequencing in Different Tissues and under Low-Temperature Stress

**Figure 10:**
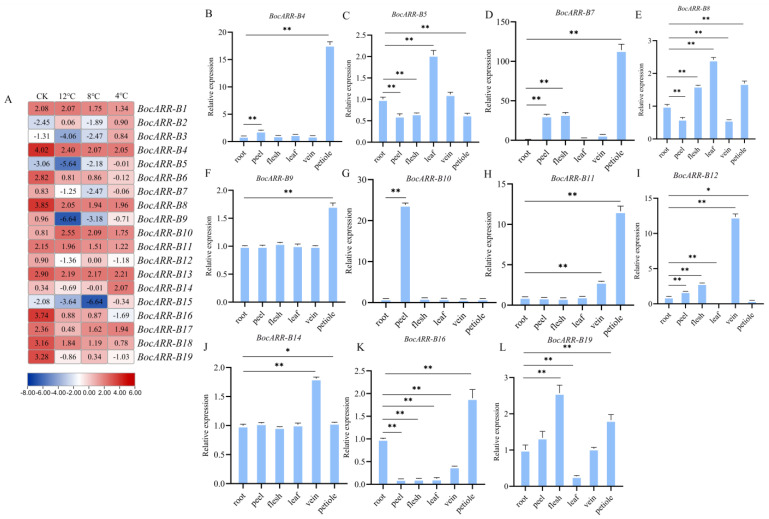
Expression analysis of *BocARR-B* family genes in kohlrabi. (**A**) Expression profile of *BocARR-B* genes in kohlrabi using RNA sequencing during low-temperature stress. Data in the heatmap box are normalized expression levels of three replicates. (**B**–**L**) RT-qPCR analysis of eleven *BocARR-B* transcription factor genes in different tissues of kohlrabi. * Represents a significant difference when the *p*-value is 0.05. ** Represents a significant difference when the *p*-value is 0.01.

To identify the expression profile of *BocARR-Bs* in kohlrabi during low-temperature stress, the relative expression levels of these 19 *BocARR-B* genes were also analyzed; the results are shown in Figure 10A. Four genes (*BocARR-B1*, *BocARR-B4*, *BocARR-B16*, and *BocARR-B18*) showed a significant downward trend under low-temperature stress. Because the expression levels of the eight genes were too low to be detected via qRT-PCR, we obtained the relative expression levels of 11 *BocARR-Bs* in different tissues (Figure 10B–L). The primers used are listed in Table S9. Eleven *BocARR-Bs* showed relatively lower expression levels in their roots. Two *BocARR-B* genes (*BocARR-B7* and *BocARR-B10*) showed relatively higher expression levels in their peels. Similarly, *BocARR-B7* and *BocARR-B12* showed relatively higher expression levels in their flesh, while *BocARR-B7* also showed relatively higher expression levels in its leaves. Three *BocARR-B* genes (*BocARR-B7*, *BocARR-B11*, and *BocARR-B12*) showed relatively higher expression levels in their veins. Three *BocARR-B* genes (*BocARR-B4*, *BocARR-B7*, and *BocARR-B11*) showed relatively higher expression levels in their petioles.

## 3. Discussion

Plant response to abiotic stress refers to the interaction of different stress-sensitive mechanisms [32]. The ability of plants to resist abiotic stress is related to the level of the antioxidant system [33]. The metabolism of ROS (active oxygen) was found to be limited in plants that contain H_2_O_2_, OH^−^, -OH, and O^2−^ at low temperatures [34,35,36]. Bychkov et al. [37] found that an increase in malondialdehyde could reduce the development of oxidative stress in Arabidopsis under hypothermic conditions (4 °C, 5 days). Garratt et al. [38] theorized that the primary enzyme involved in this antioxidant effect was SOD, which could scavenge the O^2−^ and simultaneously produce H_2_O_2_. Then, CAT degrades excess H_2_O_2_ to prevent peroxidative damage. Samarina et al. [39] discovered that the soluble sugar content and proline content were elevated in Caucasian tea under low-temperature stress. At the same time, many studies showed that in the process of resisting plant cold stress, proline can not only act as an osmoregulator but can also protect protein molecules and scavenge reactive oxygen in plants [40,41,42]. We found that the trends for CAT activity, SOD activity, MDA content, proline content, and soluble sugar content measured at different low-temperature conditions were basically consistent with the above results. In addition, plants can produce more soluble proteins to protect against damage under low-temperature stress. We also determined the content of soluble proteins, and the results showed that the content of soluble proteins increased with decreasing temperatures.

Positive/negative regulators play important roles in plants at low temperatures. The molecular mechanisms underlying the response to chilling stress are intricate and warrant further exploration [43,44]. Cui et al. [45] found that soluble sugar could enhance the low-temperature stress tolerance of sweet potato roots. At the same time, many studies showed that sucrose may function as a signaling molecule to interact with reactive oxygen species (ROS) and modulate the stress tolerance in plants [46,47]. To explore the changes in the regulatory mechanisms of DEG under low-temperature stress, we constructed a genetic metabolite network based on the glycolysis/gluconeogenesis and glucose metabolism pathway that included glyoxylate and dicarboxylate metabolism pathway and the butanoate metabolism pathway. By analyzing the regulatory DEG changes in the metabolic pathways, we obtained sixty-six DEGs related to low-temperature responses in kohlrabi seedlings.

Co-expression network analysis is an effective way to understand transcriptomic datasets better [48]. To date, co-expression network analysis has been used to screen numerous novel transcription factors and structural candidate genes in turnip [49], Arabidopsis [50], rice [51,52,53], switchgrass [54,55,56], and poplar [57,58]. Using co-expression network analysis of differentially expressed transcription factors and DEGs associated with biochemical indicator changes; we obtained twenty-two differentially expressed transcription factors, which contain *APRR1*, *WRKY32*, *TOE3*, *bHLH137*, *ARF17*, *MYB124*, and others. We hypothesized that these twenty-two differentially expressed transcription factors may regulate specific genes, thereby enhancing the synthetic accumulation of sugar and proline in the pathway, potentially improving low-temperature resistance.

The *ARR-B* transcription factor gene family is one of the important plant-specific transcription factor families, which play an important role in abiotic stress resistance and plant development [25,59]. Previous studies have shown that the *ARR-B* transcription factor family plays a key role in cold stress responses in rubber trees and paper mulberries [60,61]. Jeon et al. [27] reported that *type-B ARRs* are able to regulate *type-A ARRs*, improving the cold tolerance of crops. Kang et al. [28] reported that cold stress resistance can be improved by the overexpression of *ARR 22*. Wang et al. [30] found that the relative expression levels of *SlARR-B3*, *SlARR-B4*, *SlARR-B5*, *SlARR-B6*, *SlARR-B8*, and *SlARR-B9* in tomatoes peaked after a 9 h cold treatment. However, similar studies have not been conducted regarding the *BocARR-B* family in kohlrabi. In this study, using transcriptome data, we identified 19 *BocARR-B* transcription factor gene family members in kohlrabi. A low-temperature-stress-induced expression analysis of four genes (*BocARR-B1*, *BocARR-B4*, *BocARR-B16*, and *BocARR-B18*) showed a significant downtrend and demonstrated that some *BocARR-B* genes play key roles in indirect, positive responses to environmental stress. Specific expression analysis of *BocARR-Bs* in six different tissues (roots, peels, flesh, leaves, veins, and petioles) showed that eleven *BocARR-Bs* had relatively lower expression levels in their roots, while levels were higher in their veins and petioles. *BocARR-B7* showed relatively higher expression levels in its peels, flesh, leaves, veins, and petioles. Based on the analysis of *BocARR-B*’s expression characteristics, it is evident that *BocARR-B* genes play a crucial role in plant response to low-temperature stress, the effectiveness of which is contingent upon the specific response site, as well as the timing and severity of the stress.

## 4. Materials and Methods

### 4.1. Plant Material and Low-Temperature Stress Treatment

The green kohlrabi variety “C8” was used as the research material. It was supplied by the *Brassica* vegetable crop breeding team at the Academy of Agriculture and Forestry Sciences of Qinghai University. Kohlrabi seeds were sowed in soil and cultivated in a light incubator (Thermo Fisher Scientific, Waltham, MA, USA) at 22 ± 2 °C, under long-day specific conditions, a 12h light/12h dark cycle, 70% relative humidity, and a light intensity of 120 mmol m^−2^ s^−1^. After 35 days, growing seedlings were treated at different temperature points (CK (22 °C), 12 °C, 8 °C, 4 °C). Samples were collected on day 7, with three biological replicates for each treatment. Twelve individual plants were considered equivalent to one biological replicate under low-temperature conditions. The third and fourth leaves from top to bottom were sampled. Then, the samples were either used immediately or shock-frozen in liquid nitrogen and stored at −80 °C.

### 4.2. Measurement of Biochemical Indicators

To measure the biochemical indicators of kohlrabi at low temperature, a biochemical detection kit (catalase (CAT) activity detection kit (BC0205), superoxide dismutase (SOD) activity detection kit (BC0175), malondialdehyde (MDA) content detection kit (BC0025), proline (Pro) content detection kit (BC0295), soluble protein (SP) content detection kit (BC3185), soluble sugar (SS) content detection kit (BC0035)) were used. All biochemical indicator detection kits were manufactured by Beijing Solarbio Science and Technology Co., Ltd. (Beijing, China). The operation process of specific indicators refers to the instructions of the corresponding indicators of the kit. The statistical analysis of biochemical indicators was performed, and bar graphs were plotted.

### 4.3. Transcriptomic Analysis of Kohlrabi Leaves under Low-Temperature Stress Treatment

Leaves from the control and kohlrabi under low-temperature stress were collected. Total RNA was extracted, cDNA synthesis was carried out, and a cDNA library was constructed by Wuhan Metware Biotechnology Co., Ltd. (Wuhan, China). The obtained clean reads were used to assemble the reference sequence for further analysis by Trinity software (http://trinityrnaseq.sourceforge.net/, accessed on 18 July 2024) [62]. The unigene sequence was annotated using the BLAST search tool in the KEGG, NR, Swiss-Prot, GO, KOG, Trembl, and Pfam databases, respectively. The longest sequence obtained via Corset (https://code.google.com/p/corset-project/, accessed on 18 July 2024) hierarchical clustering was used as a unigene for analysis. The dataset was uploaded to the NCBI Short Read Archive (SRA) under the accession number PRJNA1051351 (SRR27183516, SRR27183515, SRR27183512) and PRJNA1131915 (SRR29752385, SRR29752384, SRR29752383, SRR29752382, SRR29752381, SRR29752380, SRR29752379, SRR29752378, SRR29752377).

### 4.4. Availability Validation of RNA-Sequencing

In order to validate the accuracy of RNA-Seq data of kohlrabi under low-temperature stress, ten DEGs were casually selected for quantitative real-time PCR (RT-qPCR) analysis. The samples were tested to verify that each sample contained three biological replicates. The primers used for RT-qPCR were designed by Primer Premier 5 software; the primers are shown in Table S10.

### 4.5. Co-Expression Network Analysis of Discriminated Low-Temperature-Related DEGs

In order to screen DEGs, we performed differential analyses. Genes with a false discovery rate (FDR) < 0.05 and |log_2_Fold| ≥ 1 were considered differentially expressed. Co-expression network analysis was generated by FPKM values of DEGs and six low-temperature-associated biochemical indicators using R package 3.16.5 [63] based on Pearson’s correlation coefficient. The qualification for the co-expressed genes was |r| > 0.8 and *p* < 0.05. The co-expression network in Cytoscape v3.8.0 was used to visualize the results [64].

### 4.6. Identification and Chromosomal Distribution of BocARR-B Transcription Factor Family Members in Kohlrabi

To identify all *ARR-B* genes in kohlrabi, the transcriptome unigene annotation file was used as a query [65]. ARR-B, as keywords, were screened in the KEGG files. The NCBI for online BLAST analyzed the CDS sequences that were searched. Then, in order to eliminate false-positive sequences, the analyzed cluster and homologous sequences were aligned using the DNAMAN 9.0 software. The conserved domain of the BocARR-B protein was identified using the NCBI-CDD database search tool. Interpro (http://www.ebi.ac.uk/interpro/, accessed on 29 June 2024) was used to confirm that all ARR-B proteins contained, at a minimum, the PF00072 and PF 00249 domains [66]. Full-length CDS sequences were discovered for 19 *BocARR-Bs*. The predicted physicochemical parameters of the BocARR-B proteins were obtained from ProtParam (http://web.expasy.org/protparam/, accessed on 1 July 2024). The conserved motifs of 19 BocARR-B protein sequences were predicted using the online tool MEME (http://meme-suite.org/meme/tools/meme, accessed on 2 July 2024) [67]. The chromosomal locations of 19 *BocARR-B* genes in kohlrabi were retrieved from the *Brassica oleracea* genome database, which visualized the chromosomal locations and the conserved motifs of 19 *BocARR-B* genes using Tbtools software V1.098 [68].

### 4.7. Phylogenetic Analysis of BocARR-B Transcription Factor Family

Using the *Arabidopsis* Information Resource database (https://www.arabidopsis.org, accessed on 3 July 2024), the ARR-B amino acid sequences of *A. thaliana* were downloaded. The protein sequences of 19 BocARR-Bs from kohlrabi and 15 AtARR-Bs from *A. thaliana* were aligned with a phylogenetic tree constructed by MEGA 11 using the neighbor-joining method (with 1000 bootstrap replicates) [69,70]. By the online Interactive Tree Of Life (http://itol.embl.de/upload.cgi, accessed on 3 July 2024), the phylogenetic trees were visualized and enhanced.

### 4.8. Quantitative Real-Time PCR (RT-qPCR)

RNA extraction was performed using a Tiangen RNAprep plant kit (Tiangen, Beijing, China); cDNA synthesis was performed using a PrimeScriptTM RT reagent Kit (TaKaRa, Tokyo, Japan), and the cDNA concentration was diluted to 200 ng/μL using RNase free water. The RT-qPCR reaction was performed using a TB Green Premix Ex Taq II Kit (Tli RNaseH Plus) (2X) (TaKaRa, Tokyo, Japan). In a 96-well plate, the RT-qPCR reaction (20 µL) was prepared with 10 µL of TB Green Premix Ex Taq II (Tli RNaseH Plus) (2X), 1 µL of cDNA, 7.4 µL of ddH2O, and 0.8 µL of each primer. The *TIP4* gene was used as an internal control. Each biological replicate contained three technical replicates. The expression of genes was analyzed using the comparative CT method (2^−ΔΔCt^) [71]. Excel 97-2003 and the TBtools software were used for statistical analysis and plotted heatmaps.

## 5. Conclusions

In this study, ninety differentially expressed genes (DEGs) in kohlrabi were identified under low-temperature stress through metabolic pathway analysis, and fifty differentially expressed transcription factors were obtained via co-expression network analysis. Via a comprehensive analysis of differentially expressed transcription factors and DEGs associated with biochemical indicator changes, twenty-two differentially expressed transcription factors were obtained, including *APRR1*, *WRKY32*, *TOE3*, *bHLH137*, *ARF17*, *MYB124*, and so forth. Subsequently, 19 *BocARR-B* genes (named *BocARR-B1*-*BocARR-B19*) were analyzed, and these genes were distributed unevenly across the seven chromosomes. Four conserved motifs were detected, and all 19 *BocARR-B* genes were divided into three groups. A relative-expression-level analysis of *BocARR-B* in kohlrabi showed distinct specificity across different tissues, with their efficacy contingent upon the specific response site and the timing and severity of the stress. This study lays a foundation and provides new insights into the low-temperature resistance mechanism and response pathways of kohlrabi. In addition, this study provides a theoretical basis for the functional analysis of 19 *BocARR-B* transcription factor gene family members.

## Figures and Tables

**Table 1:** Description of *BocARR-B* genes in kohlrabi.

Gene ID	Sequence ID	Accession Number of NCBI Reference Sequence	Length of cDNA	Number of Amino Acids	Molecular Weight	Theoretical pI	Aliphatic Index
*BocARR-B1*	Cluster-17807.114153	XM_013763325.1	1518	505	56,405.73	6.33	75.03
*BocARR-B2*	Cluster-17807.114154	XM_022718524.2	1491	496	55,418.6	6.24	75.6
*BocARR-B3*	Cluster-17807.84395	XM_013746126.1	1896	631	68,971.17	6.08	70.25
*BocARR-B4*	Cluster-17807.71963	XM_013816288.3	1899	632	69,032.27	6.17	69.68
*BocARR-B5*	Cluster-17807.7188	XM_013760760.1	1131	376	41,499.48	8.74	89.15
*BocARR-B6*	Cluster-17807.138306	XM_048748308.1	1314	437	48,873.92	8.87	88.1
*BocARR-B7*	Cluster-17807.90061	XM_013774622.1	1941	646	70,765.03	6.38	69.69
*BocARR-B8*	Cluster-17807.24114	XM_013774622.1	1941	646	70,941.29	6.48	69.4
*BocARR-B9*	Cluster-17807.138684	XM_013775692.1	1746	581	63,895.5	6.06	74.17
*BocARR-B10*	Cluster-17807.138685	XM_013775692.1	1734	577	63,465.09	6.06	74.68
*BocARR-B11*	Cluster-17807.122837	XM_013731190.1	2040	679	74,037.8	5.94	72.77
*BocARR-B12*	Cluster-17807.96507	XM_013731190.1	2043	680	73,913.57	5.87	73.51
*BocARR-B13*	Cluster-17807.119682	XM_013731190.1	2040	679	74,002.75	5.94	73.34
*BocARR-B14*	Cluster-17807.22246	XM_013736811.1	1608	535	60,256.55	5.28	80.5
*BocARR-B15*	Cluster-18118.0	XM_013736309.1	1506	501	56,851.62	5.08	81.08
*BocARR-B16*	Cluster-17807.71962	XM_013743001.1	1803	600	65,834.76	6.21	70.63
*BocARR-B17*	Cluster-17807.116306	XM_013743960.1	1767	588	65,877.4	5.78	77.6
*BocARR-B18*	Cluster-17807.137797	XM_013852208.3	1809	602	66,358.36	6.47	71.51
*BocARR-B19*	Cluster-17807.18348	XM_013852209.3	1806	601	66,152.09	6.38	70.67

## Data Availability

The dataset is available from the NCBI Short Read Archive (SRA) under accession numbers PRJNA1051351 and PRJNA1131915.

## Supplementary Materials

The following supporting information can be downloaded at https://www.mdpi.com/article/10.3390/ijms25179261/s1.
