# Supplementary figures and images for: Transcriptome-Based Screening of Candidate Low-Temperature-Associated Genes and Analysis of the BocARR-B Transcription Factor Gene Family in Kohlrabi (Brassica oleracea L. var. caulorapa L.)
Source: Int J Mol Sci. 2024 Aug 27;25(17):9261. doi: 10.3390/ijms25179261 (PMC11394831; doi:10.3390/ijms25179261)

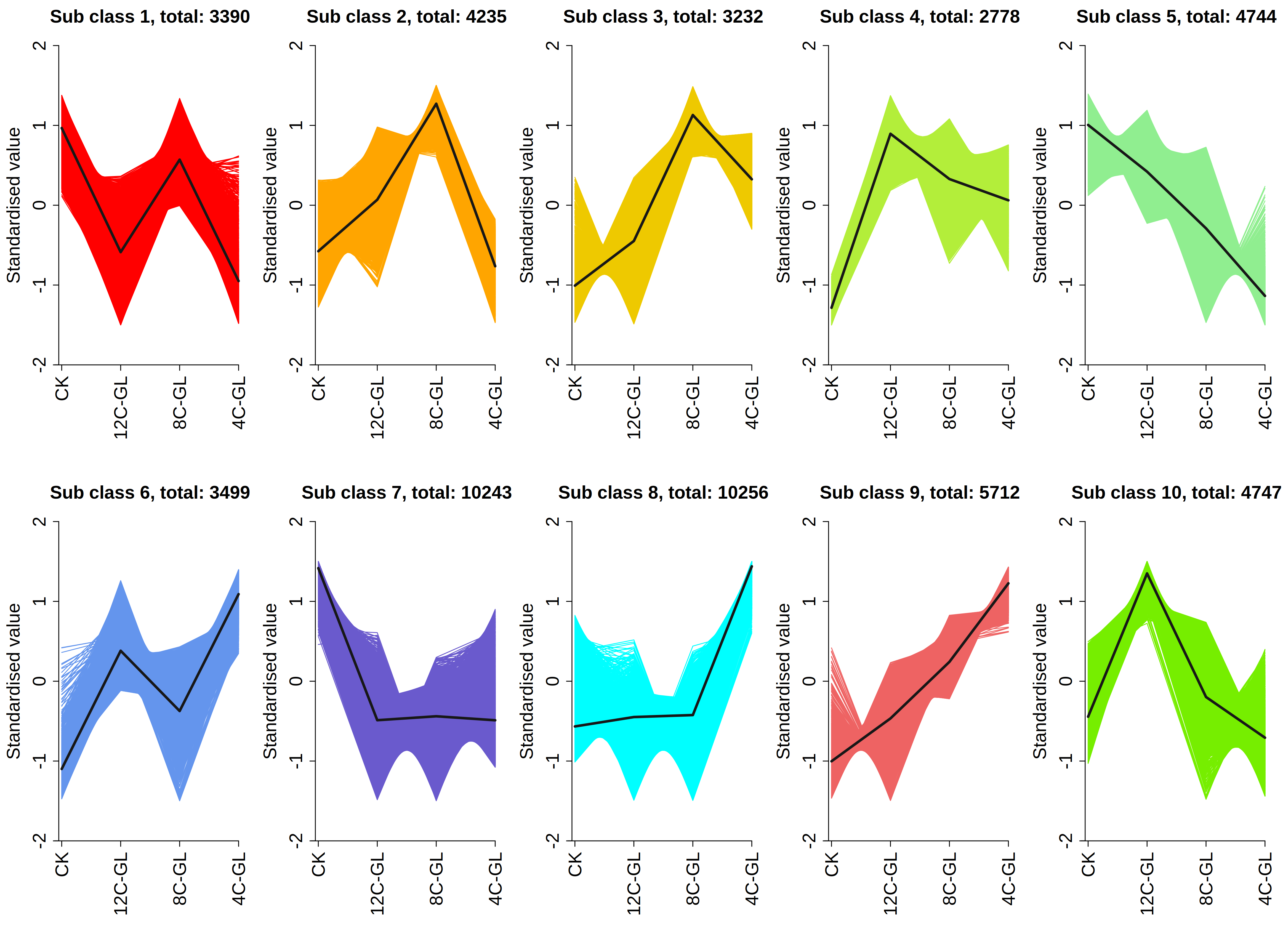

Supplement: Supplementary file 1 [file ijms-25-09261-s001.zip › Figure S2.png]

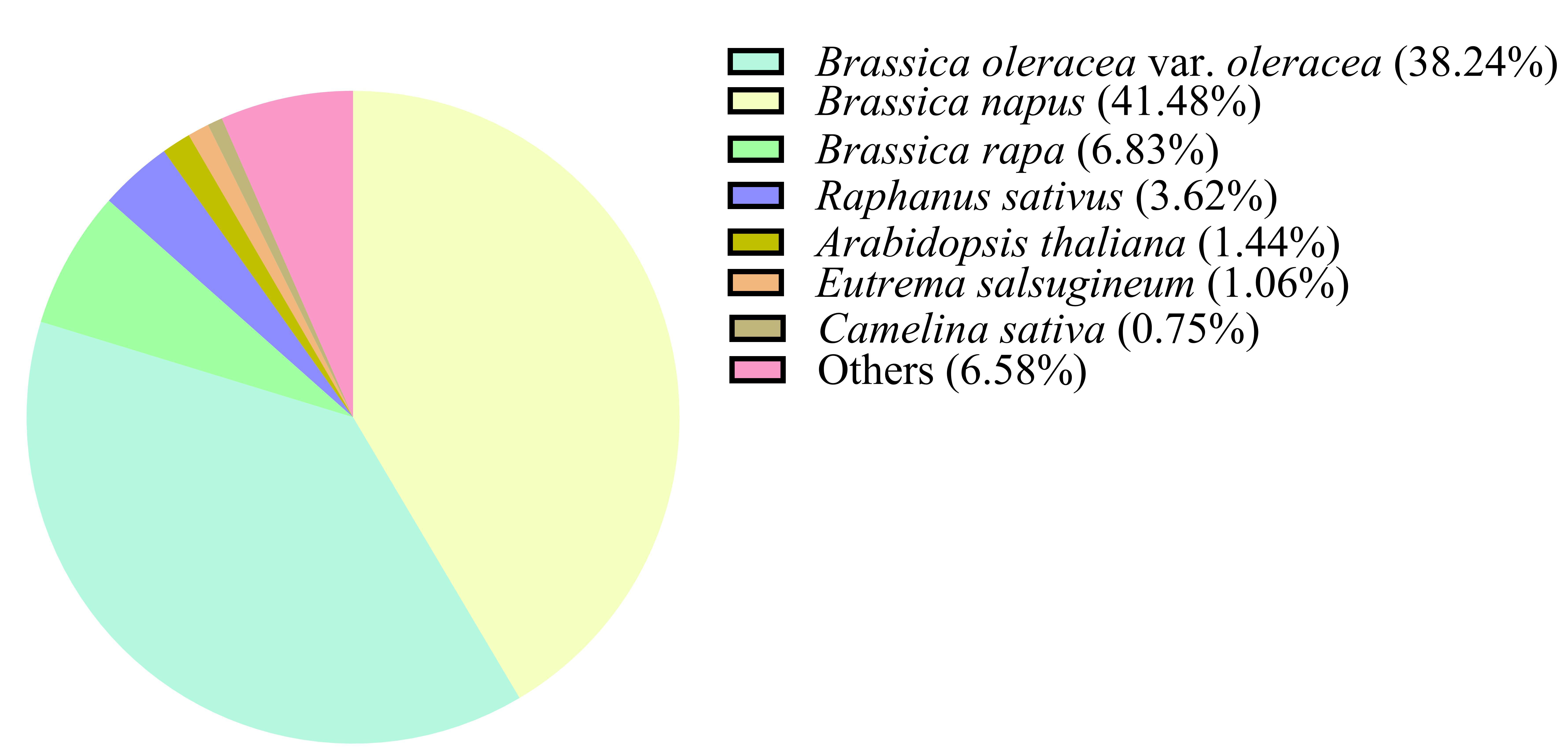

Supplement: Supplementary file 1 [file ijms-25-09261-s001.zip › Figure S3.jpg]
